# Choice of SARS-CoV-2 diagnostic test: challenges and key considerations for the future

**DOI:** 10.1080/10408363.2022.2045250

**Published:** 2022-03-15

**Authors:** Fausto Baldanti, Nirmal K. Ganguly, Guiqiang Wang, Martin Möckel, Luke A. O’Neill, Harald Renz, Carlos Eduardo dos Santos Ferreira, Kazuhiro Tateda, Barbara Van Der Pol

**Affiliations:** a Diagnostic and Pediatric Sciences, University of Pavia, Pavia, Italy; b Indian Council of Medical Research, New Delhi, India; c The Center for Liver Diseases, Peking University First Hospital, Beijing, China; d Charité – Universitätsmedizin, Berlin, Germany; e Trinity Biomedical Sciences Institute, Trinity College Dublin, Dublin, Ireland; f Institute of Laboratory Medicine and Pathobiochemistry, Molecular Diagnostics, Philipps University Marburg, University Hospital Giessen and Marburg GmbH, Giessen, Germany; g Department of Clinical Immunology and Allergology, I.M. Sechenov First Moscow State Medical University, Moscow, Russia; h Albert Einstein Hospital, São Paulo, Brazil; i Department of Microbiology and Infectious Diseases, Toho University School of Medicine, Tokyo, Japan; j Department of Medicine, Division of Infectious Diseases, University of Alabama at Birmingham, Birmingham, AL, USA

**Keywords:** SARS-CoV-2, molecular testing, PCR, point-of-care testing, antigen testing

## Abstract

A plethora of severe acute respiratory syndrome coronavirus 2 (SARS-CoV-2) diagnostic tests are available, each with different performance specifications, detection methods, and targets. This narrative review aims to summarize the diagnostic technologies available and how they are best selected to tackle SARS-CoV-2 infection as the pandemic evolves. Seven key settings have been identified where diagnostic tests are being deployed: symptomatic individuals presenting for diagnostic testing and/or treatment of COVID-19 symptoms; asymptomatic individuals accessing healthcare for planned non-COVID-19-related reasons; patients needing to access emergency care (symptom status unknown); patients being discharged from healthcare following hospitalization for COVID-19; healthy individuals in both single event settings (e.g. airports, restaurants, hotels, concerts, and sporting events) and repeat access settings (e.g. workplaces, schools, and universities); and vaccinated individuals. While molecular diagnostics remain central to SARS-CoV-2 testing strategies, we have offered some discussion on the considerations for when other tools and technologies may be useful, when centralized/point-of-care testing is appropriate, and how the various additional diagnostics can be deployed in differently resourced settings. As the pandemic evolves, molecular testing remains important for definitive diagnosis, but increasingly widespread point-of-care testing is essential to the re-opening of society.

## 1. Introduction

Since the outbreak of the coronavirus disease 2019 (COVID-19) pandemic, there has been an unprecedented effort from the scientific community to develop tools to help tackle this crisis. According to the Foundation for Innovative New Diagnostics (FIND; 23 November 2021), there are 1152 commercialized severe acute respiratory syndrome coronavirus 2 (SARS-CoV-2) assays currently available and an additional 122 in development, including 632 immunoassays and 514 molecular assays [1]. Early in the pandemic, molecular testing using nucleic acid amplification tests (NAATs) became the pillar of SARS-CoV-2 diagnostics. Since then, the development of antigen tests and immunoassays, with point-of-care and centralized options means there are now choices to be made as to how, when, and where to deploy these technologies, and many guidelines have been developed, often based on Centers for Disease Control and Prevention (CDC) and World Health Organization (WHO) guidance [2–4]. However, with the rapid emergence of new technologies and new scientific data, these guidelines are very fluid and subject to change. Furthermore, while NAATs are the most sensitive diagnostic tool for SARS-CoV-2 infection [3,4], the global demand for diagnostic testing is still such that the use of labor-intensive, specialist techniques needs to be carefully considered. This is particularly true in low-middle income countries (LMICs) where access to diagnostic tests, particularly NAATs, is limited due to a lack of medical resources, infrastructure, and trained technicians to facilitate testing [5].

The pandemic is evolving, with increasing numbers of people vaccinated, disproportionate spread of vaccination in high income countries, the emergence of new variants, and an increasing drive to return daily life to pre-pandemic patterns. The aim of this review is to provide a timely international evaluation of real-world testing needs and to define: settings where the “next best” alternatives to NAATs are appropriate; settings where NAATs may not be the best option; how to manage antigen test results; when point-of-care testing is needed, or where centralized testing can be utilized, and how to manage negative NAAT results where there is still a strong clinical suspicion of SARS-CoV-2 infection. We will further aim to set out the key considerations for defining a testing strategy. Table 1 demonstrates that each testing strategy provides different information on infection status and has different performance metrics, so the right option for the right setting needs to be carefully assessed. Here, we also discuss the common challenges facing clinicians and laboratorians when interpreting and supplying SARS-CoV-2 diagnostics and provide insights into what will be needed next.

**Table 1.** A summary of the diagnostic testing methodologies for COVID-19.

	Measure	Platforms/technologies	Turnaround time (range)	Number of samples per run/test	Performance range LOD sensitivity/specificity (%)
NAATs for viral RNA antigen detection (NP swab, oropharyngeal swab, nasal swab, sputum, bronchoalveolar lavage fluid, others)	Direct detection of SARS-CoV-2 viral RNA	High-throughput RT-PCR [6]	1.5–8 hours	Up to 384	>1.23 cp/μL [7] 450–540,000 NDU/mL [8] >90%/up to 100%
		Point-of-care RT-PCR	20 min	1	>12 cp/mL [7]
		High-throughput TMA	3 hours	Unconfirmed	600 NDU/mL [9]
		Point-of-care LAMP	20–60 min	1	>10 cp/μL [7] >75% sensitivity [10]
		High-throughput LAMP (fluorescence)	45 min	96	>1 cp/μL
		CRISPR/LAMP lateral flow	15 min	1	>6.75 cp/μL [7]
Antigen detection (saliva, NP swab)	Immunoassays for the detection of SARS-CoV-2 viral antigens	High-throughput centralized	From 18 min	Up to 300	Sensitivity (95% CI) <5 days post symptom onset and Ct <30: 97.5% (92.8–99.5%), Ct >30: 26.7% (12.3–45.9%) [11]
		Point-of-care (lateral flow)	15–30 min	1	Sensitivity (95% CI): 28.9% (16.4–44.3) to 98.3% (91.1–99.7) Specificity (95% CI): 92.4% (87.4–95.9) to 100% (99.7–100) [12]
Antibody detection (serum, plasma)	Detection of immune response, i.e. past exposure to SARS-CoV-2	High-throughput centralized	First results from 18 min to 24 hours	Up to 500	Typically, >90% sensitive and >95% specific [13]
		Point-of-care (lateral flow)	15 min	1	Typically, >90% sensitive and >95% specific [13]

CI: confidence interval; cp: copies; CRISPR: clustered regularly interspaced short palindromic repeats; Ct: cycle threshold; LAMP: isothermal loop-mediated amplification; NAAT: nucleic acid amplification test; NDU: NAAT detectable units; NP: nasalpharyngeal; RNA: ribonucleic acid; RT-PCR: reverse transcription polymerase chain reaction; TMA: transcription-mediated amplification.

### 1.1. Available classes of diagnostic tests

#### 1.1.1. Nucleic acid amplification tests

In September 2020, the WHO set out their target product profiles for SARS-CoV-2 diagnostics, stating that only NAATs are recommended for confirmation of SARS-CoV-2 infection [14]. Most NAATs have been based on reverse transcription polymerase chain reaction (RT-PCR) [8,14,15]. Transcription-mediated amplification (TMA) is another technique used interchangeably with RT-PCR [16].

Loop-mediated isothermal amplification (LAMP) is a NAAT that utilizes an isothermal reaction that does not require the thermocycling process of RT-PCR [17–19]. Studies indicate that the LAMP technique is as highly specific as RT-PCR-based technologies but reports of sensitivities vary, with some studies reporting low sensitivity kits being marketed to developing countries [19–22]. However, LAMP can be performed with minimal equipment and has been deployed to supplement widescale testing and/or where resources are limited [23].

#### 1.1.2. Antigen tests

Antigen tests are typically immunoassays designed to detect SARS-CoV-2 proteins and require no amplification. As a result, these assays often require less instrumentation and can be performed rapidly, often in near-patient settings rather than laboratories [24,25]. This class of tests may allow patients to self-sample and supports high-throughput testing [24–27]. However, antigen tests offer reduced sensitivity compared with NAATs, so adoption of these tests needs to be appropriate to the needs of the patient population served or the defined use-case (e.g. screening for same-day travel) [28]. Antigen tests detect viral proteins in a patient’s saliva or nasopharyngeal swabs, and while they have lower sensitivity than NAATs, they are most sensitive when viral loads are high, which may correlate with infectivity [29].

#### 1.1.3. Antibody tests

While NAATs are capable of diagnosing current infection, antibody testing identifies exposure to the pathogen over the patient’s lifetime, supporting diagnosis later during the disease course [30]. Antibody testing aids our understanding of SARS-CoV-2 infection and our immune response [31–34], the spread of infection [14,35–37], and, more recently, our response to vaccine administration and long-term efficacy [38]. However, as there is a delay between infection and antibody development and the presence of antibodies following recovery from infection is anticipated, recent guidance notes that antibody testing does not replace virologic testing to establish the presence or absence of acute SARS-CoV-2 infection in the majority of settings [39].

#### 1.1.4. Clinical assessments

In symptomatic patients, who may have a negative NAAT but whose clinical presentation is highly suggestive of SARS-CoV-2 and a diagnosis is required to enable medical care, a low-dose chest-computed tomography (CT) scan could be used to diagnose or rule out COVID-19 pathophysiology [40–42]. However, this is recommended with caution, as chest-CT scans are less sensitive than NAATs for SARS-CoV-2, and specificity is often over-estimated due to selection bias and the low prevalence of other pulmonary diseases in retrospective studies. The data suggest that chest-CT scans can be used to complement diagnostic testing but are not an effective standalone assessment [40,41].

## 2. Testing and sampling formats

Centralized testing is available for NAAT, antigen, and serology assays and can support high testing volumes; however, centralized testing often means longer time to results compared with point-of-care and rapid test options (Table 1). Centralized testing also requires levels of infrastructure for collection of samples and reporting of results that may not be possible in healthcare settings. Point-of-care testing provides greater flexibility and shorter time to results; it also includes automated platforms that provide rapid results and lateral flow tests.

Where high testing volumes are required but resources are limited, pooling of samples can be considered. In some settings, such as LMICs, many laboratories have adopted sample pooling strategies that allow conservation of resources [43–46]. Several commercially available NAATs have regulatory authorization for pooling and offer guidance regarding the optimal number of samples to pool and the volume per sample to include in the pool [45,47,48]. The methods and benefits of pooling are highly influenced by the prevalence in the population being tested: as the prevalence increases, pooling becomes less effective. Pooling strategies must be evaluated at each laboratory based on the population(s) they serve to minimize time to results and maximize reagent conservation [49–51], as well as to ensure the strategy remains cost-effective. However, it must be noted that pooling is a complex strategy and should be implemented with caution.

## 3. Different testing settings

In this review, we will focus our discussion on seven key testing settings: symptomatic individuals presenting for diagnostic testing and/or treatment of COVID-19 symptoms; asymptomatic individuals accessing healthcare for planned non-COVID-19-related reasons; patients needing to access emergency care (symptom status unknown); patients being discharged from healthcare following hospitalization for COVID-19; healthy individuals in both single event settings (e.g. airports, restaurants, hotels, concerts, and sporting events) and repeat access settings (e.g. workplaces, schools, and universities); and vaccinated individuals. These seven settings comprise the key areas where testing is frequently used to care for patients and help prevent the spread of infection and echo the list provided by the WHO [3].

Before diagnostic testing is considered in any individual, it is important to establish if they have symptoms and, if so, the time from symptom onset [2,4,52]. On an individual basis, these simple factors will be key to determining the relevant test choice as, for example, antigen testing may not be beneficial >10 days post symptom onset [53,54].

The considerations for the selection of a test will vary depending on the different clinical or community testing settings. The main concepts that need to be considered are:

- *Sensitivity requirements:* What is the likelihood of positive infection in the testing population and what is the risk of a false negative test? What is the likelihood of new variants/what is the need to monitor for new variants? What is the likelihood variants could result in false-positive or false-negative results? Is it necessary to detect all infected individuals or just those that are highly infectious? Is identifying the “most infectious” cases acceptable, or is the risk of missed cases high? Will other measures, such as mask wearing and social distancing, be possible in this setting?
- *Clinical/infection control considerations:* How soon are results required? What is an appropriate turnaround time from test to result? In what setting is testing/sample collection carried out? Are individuals able to quarantine while they wait for results?
- *Resource considerations:* What diagnostic resources are available and what is the priority for those resources (e.g. protecting healthcare workers, keeping schools open, diagnosing symptomatic individuals)? What scale of testing is possible in each setting? Is reliable testing feasible? If self-swabs are used, how is the quality of the sample confirmed, or will swabbing be supervised? What is the prevalence of infection?
- *Population issues (e.g. if the population is asymptomatic):* What is the local prevalence of infection? What is the likelihood of a positive result indicating that a patient is infectious? Are people attending the testing site from higher prevalence regions? This is particularly important as testing the general population may result in a high number of false-positive results [55]. What is the local vaccination update and likely time since last dose? What is the likelihood of other respiratory infections such as influenza at the time of COVID testing?

### 3.1. Testing symptomatic patients presenting for diagnostic testing and/or treatment of COVID-19 symptoms

Testing of symptomatic individuals is paramount in controlling the spread of SARS-CoV-2 infection; this is important regardless of local vaccination levels, as vaccination does not preclude infection and transmission [56,57]. The key determinants of the test for use in symptomatic patients include the patient’s symptoms/clinical presentation; whether the patient needs to be admitted for their symptoms or can manage their symptoms at home with isolation; and the setting in which patients are accessing testing/sampling and presenting to the healthcare system, local resource availability and cost-effectiveness of measure [58]. Globally, there are vast differences in how and where symptomatic individuals access healthcare, such as walk-in/fever clinics, drive-through testing centers, at-home testing squads, postal testing, and in the hospital/emergency department (ED)/general (not COVID-specific) clinic/COVID-specific clinic. If patients are accessing testing in a setting where they could possibly pass infection on to others, strict hygiene measures need to be applied and sample collection needs to be done as quickly as possible. If patients do not require urgent admission, then centralized testing is acceptable.

If patients need urgent medical care for their symptoms, then rapid testing at the point of care should be deployed so that patients are triaged as swiftly as possible. Additionally, rapid testing should be considered for testing symptomatic vulnerable populations who are unable to self-isolate while awaiting test results. Rapid tests for active infection are available as NAATs and antigen tests. If available and affordable, NAATs offer advantages over antigen-based assays, including increased sensitivity with the ability to detect patients with lower viral loads [26,27] and the potential to inform clinical stratification by means of cycle threshold (Ct) values [59–61]. In symptomatic individuals, test sensitivity is important to ensure that infectious individuals are not missed and do not continue to spread their infection, while also ensuring that those who need medical care are appropriately triaged. While the implications of false-negative results are clear, false-positives can also be problematic, leading to an overestimation of both the incidence of SARS-CoV-2 infection and the extent of asymptomatic infection, and these effects are accentuated in low-prevalence settings [62,63]. False-positives can also increase the demand on track and trace systems, and lead to people isolating unnecessarily [62]. Nevertheless, repeat testing of all positive results to identify potential false-positives is clearly impractical in the vast majority of settings.

Ct values could potentially provide a guide to early risk stratification in symptomatic patients presenting for treatment [59,60]. Low Ct values correlate with higher viral loads, with a three-point decrease in Ct values representing an approximately 10-fold increase in viral genetic material [64,65]; studies have also indicated that lower Ct values are associated with increased SARS-CoV-2 infectivity in cell culture [65]. Low Ct values could therefore be used to identify patients more likely to require intensive inpatient treatment and monitoring: a recent systematic review and meta-analysis has suggested that lower Ct values, particularly those <25, are associated with more severe disease requiring ventilation or admission to intensive care, as well as higher mortality [60]. Of note is that the timing of specimen collection may impact the utility of these values: early in the course of infection, Ct values are generally low, and may not be useful as a surrogate marker of disease severity [60]. By contrast, Ct values may have clinical utility in patients whose symptoms progress as a result of persistently high viral loads, and these are the patients most likely to present in the healthcare setting [60]. There are, however, a number of other limitations to the routine use of Ct values in clinical care. Not all NAATs provide Ct values and, for those that do, there is variability in the results obtained between platforms, between labs, and between reagent lots within a single lab [61]. This is because Ct can be affected by collection technique, specimen type, sampling time, viral kinetics, transport and storage conditions, nucleic acid extraction, viral RNA load, primer design, real-time PCR efficiency, and Ct value determination method [66]. As such, Ct can provide clinical guidance only once standardization practices for estimating viral concentration from Ct values are in place.

**Figure 1.**
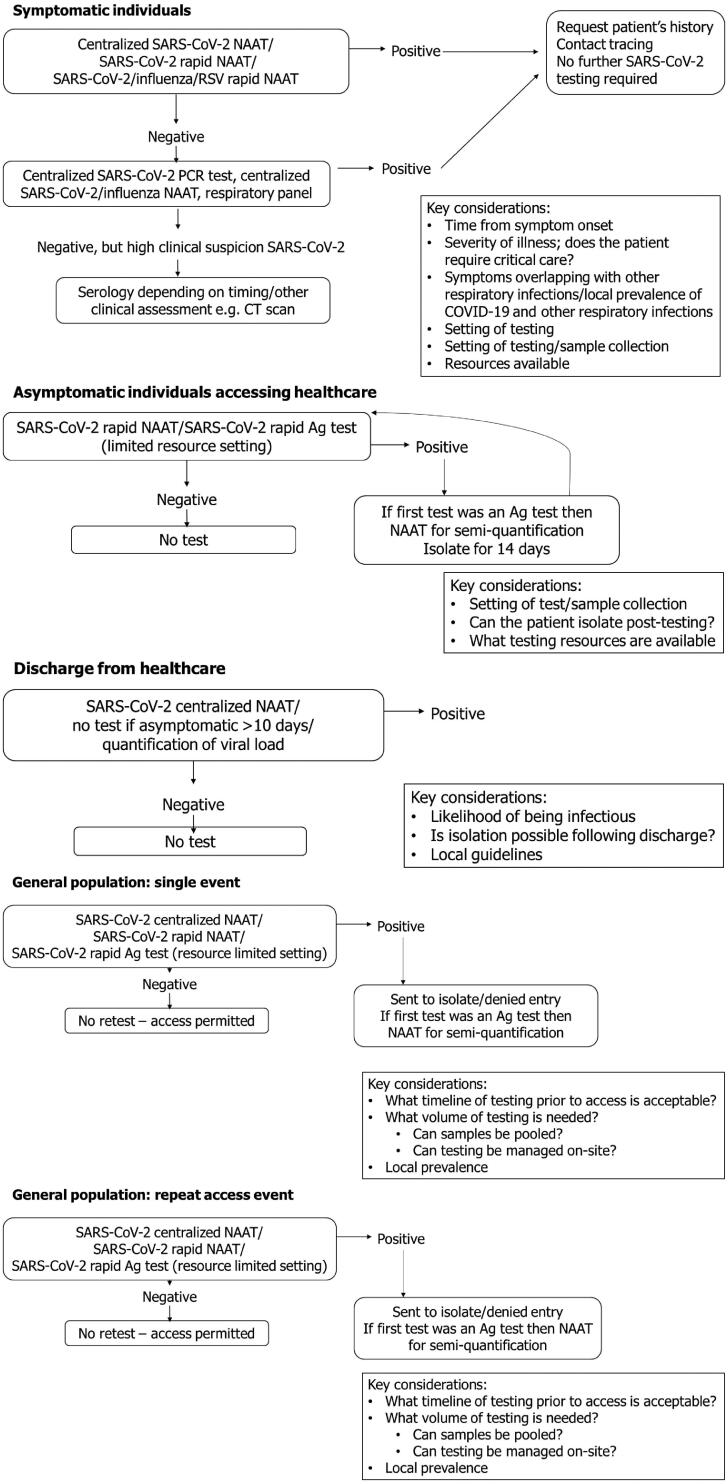
Testing strategies and considerations for the different diagnostic settings considered in this publication. Ag: antigen; CT: computed tomography; NAAT: nucleic acid amplification test; PCR: polymerase chain reaction.

The priority for symptomatic patients is the need to know if their symptoms are due to SARS-CoV-2 infection; in these cases, NAATs for SARS-CoV-2 should be performed when possible (Figure 1). NAATs are the most sensitive class of tests available, and this method will help to ensure that cases are not missed among symptomatic patients [26,67]. In this context, RT-PCR testing and TMA are appropriate compared with LAMP, as sensitivity data are still variable (Table 1) [17–20,23,68–70].

Limits to resources available for PCR testing are heightened in LMICs with high-prevalence of SARS-CoV-2 where there are a lack of trained staff, finances, and infrastructure, including biosafety level 2 laboratories, therefore alternative testing strategies will also be needed. In settings where access to NAATs is limited or turnaround times are too long [5,71,72], antigen testing is also an acceptable option for the diagnosis of symptomatic patients, as it is more informative than no testing. Total and IgM-based antibody tests have also been shown to have some diagnostic benefit, in the absence of NAAT/antigen tests for acute infection [73,74]. For example, rapid antigen-based and antibody-based diagnostic tests have shown promising results in Cameroon although the diagnostic value of antibody-based tests may be limited in the early stages of disease [75]; in the first seven days after symptom onset, antigen test sensitivity was 80.0% whereas antibody test sensitivity was only 26.8%; however, this increased to 76.4% 14 days after symptom onset [75]. These sensitivities are lower than the “gold standard” ≥98% test sensitivity considered “desirable” by the WHO [14]. Guidelines, therefore, suggest that antibody tests should not replace NAAT/antigen testing where these are available [39]; however, antibody tests may have some value in resource-limited settings [75]. In India, an antigen-based rapid diagnostic test has shown 85.9% sensitivity at detecting SARS-CoV-2 in patients with a duration of illness ≤5 days [76].

If symptoms are strongly indicative of SARS-CoV-2 infection, a negative antigen test should also be confirmed with a NAAT [77–80]. The authors consider that specificity is not an issue with currently available antigen tests, and that while retesting is not needed to confirm positivity, NAATs may be performed to provide semi-quantitative Ct values to aid understanding of infection status [26,28,78,80–82]. However, as noted above, the utility of Ct values is currently unclear and the use of Ct values to assess infection status is currently only deployed in certain regions, and only then in patients who require medical intervention for COVID-19.

Depending on the local prevalence and patient-specific risk of influenza, dual-target NAATs for influenza and SARS-CoV-2 infection may be useful for differential diagnosis, particularly if an initial NAAT result is negative and clinical suspicion of respiratory infection is high (Figure 1). In many regions, the prevalence of influenza has been very low, possibly due to infection control measures for SARS-CoV-2, with a lower than normal risk compared with that expected for many regional flu seasons [83–87]. However, in populations more heavily vaccinated against SARS-CoV-2, the influenza risk is anticipated to increase as society begins to open up. In this regard, it is important to note that SARS-CoV-2/influenza coinfection can occur leading to increased risk of death compared with that associated with either virus alone [88].

Digital screening tools are also in development to track COVID-19 symptoms and support the identification of infection. In Brazil, where testing resources are limited, an app-based symptom tracker has been developed to help prioritize those who should be tested [89]. After implementation of the app, the proportion of positive results increased significantly from 14.9% to 18.1%.

### 3.2. Testing asymptomatic patients accessing healthcare

#### 3.2.1. Testing asymptomatic patients prior to hospital admissions for planned non-COVID-19-related reasons

Patients in this category would need testing prior to hospital admission in order to prevent nosocomial SARS-CoV-2 transmission and to triage patients appropriately within the hospital setting [90,91]. These patients would not have symptoms of COVID-19 and would be attending routine healthcare appointments or planned surgeries; these appointments are not considered urgent care.

Point-of-care or centralized NAATs are generally used to test patients prior to admission, as NAATs are the most sensitive method for detection of SARS-CoV-2 and would maximally prevent the spread of infection to the healthcare system. Patients may be able to isolate while they await their test results, making centralized testing possible in some settings. The urgency of the care that is required also determines whether the patient should have a rapid point-of-care test or whether a centralized option would be acceptable. As patients are asymptomatic, there is no need for repeat testing; if they test positive, they should isolate for 14 days and then retest for SARS-CoV-2 infection [91]. A disadvantage of using NAATs in this setting is that studies have shown prolonged NAAT positivity in patients who are no longer symptomatic following infection, and it may be that noninfectious virus is detected [29,92–94].

As these patients are asymptomatic, antigen tests may not be adequately sensitive to detect SARS-CoV-2 infection (Table 1) [4,79,95]. In this setting, if patients have a positive antigen test result they should be treated similarly to patients with a positive NAAT, requiring them to isolate for 14 days and retest (Figure 1). Despite antigen sensitivity being quite low in asymptomatic people, the use of rapid antigen testing in areas with a lack of resources for NAAT testing should be considered to help prioritize those who should be tested further. The risk of false-negative tests is currently mitigated by the universal SARS-CoV-2 precautions utilized in healthcare settings and will be further mitigated by the increasing vaccination coverage of the healthcare workforce. If clear information emerges indicating the sensitivity of antigen tests in detecting *infectious* individuals, widespread use of these tests could be mandated in order to prevent unnecessary delays to planned appointments. More information regarding how diagnostic tests relate to infectivity is needed before this is possible.

#### 3.2.2. Testing patients requiring urgent hospital admission, asymptomatic/symptom status unknown

The considerations in this setting are the same as outlined above for routine admissions; however, in these patients, urgent care is needed and point-of-care NAATs should be deployed to provide swift and accurate results (Figure 1). These patients are not being admitted due to symptoms of COVID-19 and, as such, repeat testing following a negative NAAT test would not be required, unless indicators arise to suggest a patient does have respiratory symptoms.

If results can be obtained more quickly using an antigen test, then this is an acceptable stop-gap before a NAAT result can be confirmed, which might be performed on-site in a centralized laboratory [90]. In patients with respiratory symptoms in the ED setting, antigen testing has still been shown to produce false-negative results [90]. Depending on the clinical setting and the care that the patient requires, other assessments for the presenting condition may also reveal the likelihood of a respiratory infection but are not diagnostic for SARS-CoV-2 infection [96].

### 3.3. Patients being discharged from healthcare following treatment for COVID-19

COVID-19 patients are tested prior to discharge to ensure they are not infectious. Globally, the approach to managing patients leaving healthcare following treatment for COVID-19 is variable. In the United States (US) and Japan, patients are expected to be symptom-free for a period of at least 10 days; in Germany, patients must have a Ct value >30 for discharge to nursing homes; in Italy, absence of symptoms and a negative NAAT are required for discharge [97–99]. Other countries only require a negative NAAT if the patient was severely unwell (e.g. receiving supportive oxygen) [97]. In China, patients are discharged if they are no longer symptomatic and have a repeated negative NAAT for SARS-CoV-2 within 24 h [97]. Chinese patients are then required to isolate for a further 14 days and may be discharged to an interim/recovery hospital for further isolation and monitoring before returning home [93]. These stringent criteria are due to reports of relapsing infection and aim to prevent transmissions in these cases [100].

The challenge of using NAAT in this setting is that particular patient populations, such as post-transplant and/or immunocompromised patients, stay PCR-positive for a longer time period than in the general population [101]. Assessment of viral culture from recovered patients indicates that PCR positivity post-infection does not correlate with the presence of infectious virus [102]. Ct values can provide a guide to infection status in these individuals: a recovered patient with a high Ct will have a low viral load and is unlikely to be infectious [61,103]. However, due to the limitations of Ct values discussed earlier, they should be interpreted with caution and any interpretation should be done in the context of the clinical case [104].

In the future, quantification of viral load and standardization of Ct values may be widely applicable, aiding determination of infectious periods and possibly reducing the duration of hospitalization for some patient populations [105]. In patients who remain NAAT-positive for a prolonged time period, antigen testing may better reflect if a patient is still infectious, as previously described above.

### 3.4. Testing asymptomatic individuals in the general population

#### 3.4.1. Single event settings

This category encompasses a broad range of scenarios and recommendations will need to be specific for each setting. There is no standardized approach to testing within the community, both NAATs and rapid antigen tests are being widely used and the standard is often driven by businesses such as offices, airports, and restaurants. The drivers for single access testing will include the number of individuals attending the event, local prevalence of infection, if social distancing and hygiene-based infection control measures can be maintained, what would be the impact of a positive case in that setting, and the feasibility and scale of testing needed for the event. Vaccination status is also important: as more people are vaccinated, their status can be proven by certificate (and possibly also an antibody test result), and this may be required to access alongside a negative antigen test. Centralized NAATs could be used to deliver high volumes of testing and could allow sampling to occur at remote sites prior to access (e.g. people accessing an airport or a stadium event). In this asymptomatic population, pooling samples could be useful in order to maximize the testing capacity, although this may reduce the sensitivity of tests and, as such, is most appropriate for use with centralized PCR testing [43,49–51,106]. The expense and time-to-result would not be practical for activities such as visiting a shopping center or restaurant. Highly sensitive antigen or NAAT point-of-care tests could be performed by non-laboratory trained personnel in these settings; however, while these tests provide quick results (15–90 min), they may not be suitable to conduct in crowded environments. Lateral flow antigen tests could be a simple and cost-effective way to test large groups of people; however, studies report high numbers of false-negative and false-positive results, with potentially important implications for transmission risk, meaning SARS-CoV-2 infection precautions (masking and social distancing) should ideally still be employed [28,55,107]. Initial findings from the Events Research Programme in the UK identified only 28 positive cases of SARS-CoV-2 infection among >58,000 people attending nine events in 2021 for which a negative lateral flow antigen test was required as a condition of entry [108,109]. However, only 15% of participants also had a NAAT before and after the event, decreasing the robustness of the evidence, and the design of the study meant that it was not possible to directly attribute infection to attending the event itself [108,109]. In addition, the events were a diverse mix of settings with a range of infection control measures in place, further complicating interpretation of the results [108,109].

As vaccination rates increase and countries steadily begin to open up, SARS-CoV-2 testing has become an integral part of strategies to allow international travel. Even in those individuals with proof of vaccination, many countries require proof of a negative SARS-CoV-2 before travel to that country and/or following entry [110,111]. While the exact requirements differ by country, several countries are moving away from the requirement for NAAT testing toward the acceptance of rapid antigen test results (sometimes with NAAT for confirmation of a positive result); antibody testing is generally not considered an acceptable method to preclude active infection [110,111].

#### 3.4.2. Repeat access settings

Repeat access settings comprise workplaces, universities, schools, and hospitals, where the same group of people repeatedly interact together. Testing in repeat access settings is already being widely conducted, for example, many hospitals are regularly testing their healthcare staff using NAATs [112]. In healthcare staff, regular testing is leading to the identification of many SARS-CoV-2 cases, enabling prompt isolation and therefore limiting outbreaks within hospitals [113]. In healthcare settings, testing staff has clear benefits with respect to the costs involved with screening, namely the prevention of SARS-CoV-2 outbreaks among hospital staff, subsequent staff absences, and nosocomial transmission to potentially vulnerable patients. Pooling samples can also help to make screening these populations more resource efficient, particularly in LMICs where resources might be limited [43,106].

During the peak of the COVID-19 pandemic, when case numbers were very high, professional athletes and their support staff around the world have been subject to regular testing so that elite sports can continue during the pandemic. This is often supported by isolation, social distancing, personal protective equipment for staff, and other measures to prevent infection [114–116]. In these professional settings, the funding is often available to test regularly to ensure that the sports continue to operate, and these decisions sit with the sporting bodies and national governments. As increasing numbers of individuals are vaccinated and case numbers continue to decline in many countries, these stringent measures may no longer be necessary and may only apply to single access events such as sporting competitions, particularly those involving international travel.

For schools and universities and most non-medical workplace settings, NAATs may not be needed or have the appropriate cost–benefit, as antigen testing or LAMP may be sufficient to detect the most infectious cases. In addition, older students may be able to adhere to some social distancing and mask-wearing measures. Overall, transmission has been noted to be lower in younger pupils compared with older pupils [117,118]. Rapid antigen tests can allow for regular at-home testing, potentially reducing the need for very high sensitivity.

In these repeat access settings, a single infection could become an outbreak. Importantly, the extent of vaccination coverage, local prevalence of infection, and necessity of the contact should be carefully considered before allowing any gathering of individuals. Even in high-risk individuals such as those in care homes, SARS-CoV-2 vaccination has been associated with significant reductions in infection rates, and significantly lower risk of morbidity and mortality following SARS-CoV-2 infection compared with those seen pre-vaccination [119,120].

### 3.5. Testing vaccinated individuals

Several vaccines have now been shown to provide protection against COVID-19 [121,122]. There are still many unanswered questions regarding the longevity of immunity offered by vaccines: if they will be efficacious against all strains and variants of SARS-CoV-2, which vaccines are most efficacious in different patient cohorts, and the need for and timing of booster doses, particularly in those people considered “high risk”. Studies are ongoing to answer all these questions using a range of testing strategies.

SARS-CoV-2 infection can result in antibody development against viral proteins including the spike (S) and nucleocapsid (N) proteins, with 90–99% of individuals developing detectable neutralizing antibodies within 4 weeks of infection [123]. By contrast, SARS-CoV-2 vaccines have generally been designed to elicit an antibody response against the S protein. In vaccinated individuals, anti-N antibody tests have been used to determine prior infection [124,125]. In order to assess the longevity of vaccine-mediated immunity, high-throughput quantitative anti-S antibody tests are likely to be useful [126]. Many serological assays have been shown to correlate with neutralizing antibody titers [127,128]; however, direct assessment of neutralizing antibodies may be preferable where possible as it is not fully understood how antibody test positivity relates to protective immunity against SARS-CoV-2 [129,130]. In a meta-analysis of phase 3 vaccine trials and neutralization titers in convalescent patients, a significant association between vaccine efficacy and neutralizing antibody titers has been reported at the study level [131] but comparison of the immune responses provided by different vaccines is challenging as vaccine developers have used a range of different approaches to assess immunogenicity [131,132]. In order to harmonize the reporting of different assays, WHO International Standards for anti-SARS-CoV-2 immunoglobulin have been developed allowing the standardized reporting of neutralizing activity in international units (IU)/mL and binding assays in binding antibody units (BAU)/mL [132]. In a recent analysis of data from vaccinated individuals reporting results using these standards, direct correlations between higher anti-S IgG, anti-receptor binding domain (RBD) IgG, and neutralizing antibody titers with lower risk of symptomatic disease were observed [133]. With the emergence of new strains and availability of new vaccines, serological assays may, therefore, be useful to inform future behaviors such as the need for and timing of booster doses and the choice of vaccine in different populations, including those who are immunocompromised or have other comorbidities. Assessments of cellular immunity are also necessary to completely understand how SARS-CoV-2 vaccines offer protection and how long this protection lasts.

In order to assess whether new variants are emerging that have the potential to escape vaccine-mediated immunity, full genome analysis is needed to better track evolution and spread of lineages, with particular focus on regular S gene sequence analysis and vaccinated sera challenge studies of emergent strains [134,135].

## 4. What are the testing considerations for the next steps in the pandemic?

A main focus for diagnostics will now be the ongoing monitoring of emergent strains. As discussed, this will be essential to ensure that the global rollout of vaccines is successful and to help the international community emerge from the pandemic [134]. The unambiguous identification of the specific variant causing infection requires whole genome sequencing and is not possible with the diagnostic tests routinely used to identify the presence or absence of infection [136]. However, when PCR-based assays are used for diagnostic testing, the European Centre for Disease Prevention and Control recommends that confirmatory sequencing of at least a subset of viruses should be performed to use these assay results as indicators of community circulation of variants of concern [136]. In addition, for specific RT-PCR assays, their patterns of detection can be used to provide an indication of the likely presence of a particular variant which can then be followed by confirmatory sequencing [136]. For example, for the B.1.1.7/501Y.V1 variant, a negative or significantly weaker positive S-gene result in multiplex RT-PCR assays, with positive results for the other targets, has been used as an indicator of the presence of this variant (so-called “S-gene drop out” or “target failure”) [136]. Identification of a specific variant is not possible using antigen-based tests [136]. In terms of ensuring that diagnostic tests capture newly emerging strains, as most NAATs detect several SARS-CoV-2 genetic targets, it is considered unlikely that mutations will lead to false-negative results; however, the US Food and Drug Administration (FDA) has requested that laboratorians are mindful that this may occur [137]. Studies indicate that antigen tests have so far remained effective against new variants of SARS-CoV-2 [138]. Antigen tests could possibly have an advantage over some NAATs in recognizing new strains, as the primers NAATs use may not be able to detect the new mutations. It will be crucial to continually monitor the performance of diagnostic tests against emerging variants.

Standardization of Ct values or fully standardized quantitative NAATs for SARS-CoV-2 will be extremely useful to assess efficacy of interventions in COVID-19 patients, to help determine when individuals are safe to leave quarantine and when staff are safe to return to work [105]. However, developing standardized, reproducible viral load quantification assays is a challenge and this has been achieved for few viruses to date [65]. Development of reference material to enable standardization of these assays is recommended as has been done in other settings such as the development of quantitative viral standards for most widely recognized transplant-associated viruses [139].

While data regarding the relationship between diagnostic parameters and infectivity is emerging, research is ongoing in this area to clarify exactly how they correlate. Determination of the antibody titer at which protection against infection is achieved will be important to inform future decisions around choice of vaccination and/or use of booster doses [140]. Recommendations on the most appropriate diagnostic test for different utilities may change once more information is available on these points.

The use of alternative sample types to the nasal pharyngeal (NP) swab, such as saliva, and the use of new technologies, such as CRISPR-based tests, are under investigation, and these may also provide different opportunities for testing and additional considerations (e.g. those concerning school populations or mass testing of large groups) [31,70,141,142]. Self-sampling methods that provide increased sensitivity could broaden the capacity for mass testing prior to events or entering the workplace [27]. As it will take time before vaccination will be able to reduce the impact of SARS-CoV-2 infection, testing measures will continue to be important.

## Abbreviations

BAU: binding antibody units
CDC: Centers for Disease Control and Prevention
COVID-19: coronavirus disease 2019
CT: computed tomography
Ct: cycle threshold
ED: emergency department
FDA: Food and Drug Administration
FIND: Foundation for Innovative New Diagnostics
LAMP: loop-mediated isothermal amplification
LMIC: low- and middle-income country
N: nucleocapsid
NAAT: nucleic acid amplification test
RBD: receptor binding domain
RT-PCR: reverse transcription polymerase chain reaction
S: spike
SARS-CoV-2: severe acute respiratory syndrome coronavirus 2
TMA: transcription-mediated amplification
US: United States
WHO: World Health Organization
